# The indirect cost due to pulmonary Tuberculosis in patients receiving treatment in Bauchi State—Nigeria

**DOI:** 10.1186/1478-7547-10-6

**Published:** 2012-05-11

**Authors:** Nisser Ali Umar, Richard Fordham, Ibrahim Abubakar, Max Bachmann

**Affiliations:** 1 Health Economic Research Group, School of Medicine, Health Policy and Practice, University of East Anglia, Norwich, NR4 7TJ, UK; 2 Tuberculosis Section, Centre for Infections, Health Protection, Agency, London, UK; 3 Health Services Research Group, Norwich Medical School, University of East Anglia, Norwich, NR4 7TJ, UK

## Abstract

**Objective:**

To determine the time spent and income lost by patients and their households for seeking tuberculosis diagnosis and treatment in Bauchi State-Nigeria.

**Method:**

A cross sectional study where 242 TB patients were sampled from 27 out of 67 facilities providing TB services in a north-eastern state of Nigeria. Sampling was stratified based on facility type, patients’ HIV status and gender.

**Results:**

The income lost among the hospitalized group was estimated at $156/patient and about $114 in the non-hospitalized patients group. Age, gender, facility of diagnosis, level of education and occupation were significant (p-values <0.05) associated with total (both patients and their households) income lost. However, AFB sputum-smear result and HIV status had no significant effects on the income lost. Hospitalised patients spent an average time of 924.98 hours for diagnosis and treatment whereas the non-hospitalised spent an average of 141.29 hours. The estimated US dollar valued of these hours was US517.98 and US$79.13 for hospitalised and non-hospitalised patient groups respectively. Hospitalisation and the facility of diagnosis were statistically significant (p-value <0.05) predictors of the time patients and household spent on TB.

**Conclusion:**

Tuberculosis poses causes tremendous burden in terms of time and productivity lost to both patients and their households in Bauchi State Nigeria.

## Background

It has been estimated that about one-third of the world’s population are currently infected with Mycobacterium Tuberculosis and about 3 million deaths are attributable to tuberculosis each year despite the availability of antibiotics that can cure this controllable affliction [1-3].

The WHO estimated the global prevalence of active Tuberculosis at 217 per 100,000 people and incidence rate of 136 per 100,000 people in 2007 [4].

Nigeria was ranked fourth in burden of Tuberculosis (TB) globally with incidence rate of 311 per 100,000 populations, prevalence of 521 per 100,000 population and 93 mortalities due to TB per 100,000 populations in 2007 [4] implying significant social and economic burden in the country [5,6].

Several studies have assessed the patient and household out of pocket costs of TB in sub Saharan Africa and other third world countries and others have studies the cost-effectiveness of different approaches to TB treatment in many countries [7-19] but there are very few, if any, done anywhere in Nigeria, nor study that assessed the indirect costs to households, communities and patients with tuberculosis in terms of man hours spent by patients with TB or their households and the associated productivity lost.

Considering the fact that TB services are increasingly becoming reliant on informal care, shifting costs from the health care sector to the communities through early discharge programmes, substitution of inpatient care with ambulatory care and the move toward community care of tuberculosis, the need to study the indirect cost of TB becomes imperative.

### Study objective

This study is aimed to estimate the indirect cost of tuberculosis from the US dollar value of the time spent and productivity lost by patients, families and others due to tuberculosis illness in Bauchi state- Nigeria.

### Study area

Bauchi state is located in the North Eastern region of Nigeria and is the 7^th^ most populous state in the country. It occupies a land mass area of 49,259 sq Km with a total population of 4,676,465 as at 2006 population census [5].

### Setting

Tuberculosis treatment service in Bauchi state is provided by 2 tertiary hospitals, 23 general Hospitals, 1 Infectious diseases hospital, 14 primary healthcares with diagnostics (microscopy) capacity, 25 treatment centres (also primary healthcares centres) and 2 privately owned clinics [6]. Direct Observation Therapy (DOT) widely acclaimed by most facilities to be the standard practice for the treatment of TB in the state [6].

### Study design and methods

This is a cross sectional study in which the time spent by patients and other household members for tuberculosis (TB) diagnosis and treatment was assessed as well as the income lost (both the patients and households) due to the tuberculosis illness.

A total of 242 (initially 255 but 13 were excluded based on age criteria of less than 15 years or older than 59 years of age) TB patients were sampled from 27 out of 67 facilities providing TB services in the state. The sample size was allocated based on facility type and patients were randomly selected in each facility. Selection was stratified based on patients’ HIV status and patient’s gender. The stratification was done during the selection and where randomly selected number of patients in a stratum got at least half of the allocated sample size the subsequent random selections will only be valid if is for the other stratum. is A total of 40 patients were selected from the Infectious Disease Hospital, 40 from the Specialist hospital (tertiary hospital), 10 patients each from 9 General Hospitals, 6 patients each from 5 PHC diagnostic centres, 5 patients each from PHC treatment centres.

Only patients with ‘confirmed’ TB diagnosis were included. Most of these patients had at least one sputum smear positive test and few had only sputum negative results but had chest x-rays strongly suggestive of TB with history of significant clinical improvement after initiation of TB treatment.

An ethical approval was sought and granted for this research by the Bauchi State Ministry of Health. The study was conducted between May and August, 2008.

A standardized questionnaire (with the permission of the original developers [20]) was used to estimates the indirect costs of TB on patients, their families and on others for seeking and accessing TB treatment during pre-diagnostic, diagnostic and post diagnostic period as well as during hospitalisation where applicable. The questionnaires were administered to the entire patients individually.

The indirect cost in this study was estimated from:

i. The average time spent by patients, their relatives, friends and other unpaid carers on travel, waiting and time for consultation, treatment and hospitalization by TB patients and persons who accompanied patients during the period starting from the onset of illness that lead to TB diagnosis to the time TB treatment was completed. The monetary value of the time was calculated from the hourly wage value estimated at US$0.56/hr based on the 2008 annual gross national income per capita in Nigeria, which is $1170 [21]. Annual working hours per capita used in this estimate was 2080 hours (40 hours per week for 52 weeks).

And:

ii. Income lost by TB patients and their households due to TB illness or complication resulting from TB disease or treatment as estimated from the difference in self-reported monthly patients and household income in the periods before and during TB illness.

Data was entered into IBM SPSS version 19 software and descriptive data analysis as well as univariate general linear modelling for test of between subject effects of some demographic and socioeconomic variables on the total indirect cost.

## Result

About 104 (43.0%) of the patients in this studies were hospitalized within the period from 6 months before TB diagnosis through the period of TB treatment (Table 1).

**Table 1:** The characteristics of the study population

**Description**	**Number (%)**
History of hospitalization at least 6 months before diagnosis, during diagnosis and after diagnosis	
Hospitalized	104 (43.0%)
Not hospitalized	138 (57.0%)
Gender	
Female	110 (45.5%)
Male	132 (54.5%)
History of prior TB illness	
New TB cases,	218 (90.1%)
Retreatment	24 (9.9%)
Reasons for retreatment	
- Relapse,	20 (83.3% of the retreated)
- Default	2 (8.3% of the retreated )
- Treatment failure	2 (8.3% of the retreated)
HIV status	
HIV negative,	106 (43.8%)
HIV positive	122 (50.4%)
Unknown HIV status	14 (5.8%)
Employment	
Un-employed	75 (31.0%)
Students	10 (4.1%)
Civil servants	28 (11.6%)
Small scale businesses	53 (21.9%)
Farmers	40 (16.5%)
Drivers, Labourers, Security guards or Menial workers	26 (10.7%)
Commercial sex workers.	10 (4.1%)
Average annual income	
Female	$449.90/year
Male	$960.65/year
The mean age of the sample population	32.8 year (15 – 59 years)

One hundred and thirty two (54.5%) of the patients were male, average age of the sample was 32.8 (±9.8 SD) years. Only 24 (9.9%) of the patients in this study had history of previous TB infections of which 20 (83.3%) of the retreatment cases were reportedly due to relapse, 2 (8.3%) due to default and another 2 (8.3%) due to treatment failure; Only 22 (9.1%) of the patients had all sputum AFB tests negative. About 106 (43.8%) of the patients were HIV negative, 122 (50.4%) were HIV positive and 14 (5.8%) did not declared their HIV status.

Ninety three (38.4%) of the patients had no any formal education, 52 (21.5%) had primary school certificates, 18 (7.4%) had junior secondary school certificates, 60 (24.8%) had secondary school certificates, 15 (6.2%) had undergraduate certificates and 4 (1.7%) had graduate degrees and above. Seventy six (29.8%) of the patients were unemployed, 18 (7.1%) were students, 55 (21.6%) were small scale business men and women, 42 (16.5%) are farmers, 26 (10.2%) were either drivers, labourers, security guards or menial workers and 10 (3.9%) were commercial sex workers.

Average number of people living in the patient’s household was 6.43 (±5.37 SD). Average delay in diagnosis was estimated at 5.61 (±2.67 SD) weeks and the average number of facilities visited before diagnosis were 2.74.

### Income lost

**Figure 1:**
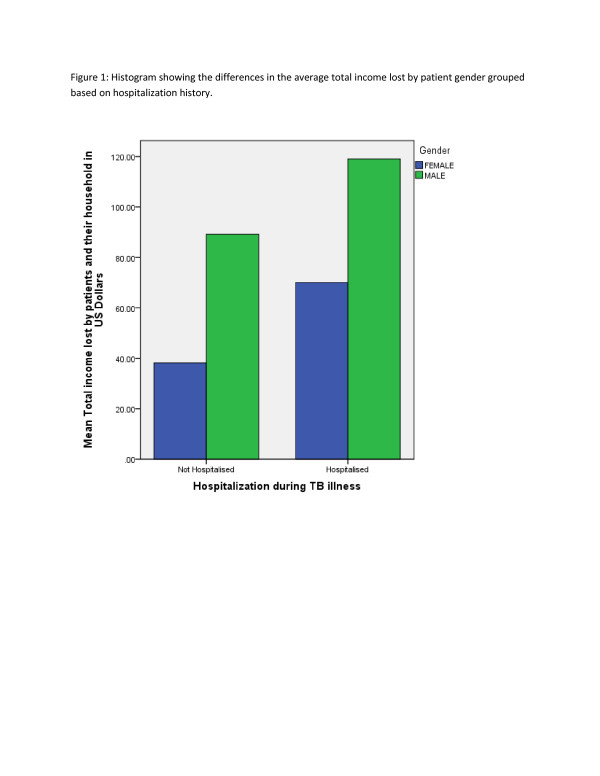
Histogram showing the differences in the average total income lost by patient gender grouped based on hospitalization history.

**Figure 2:**
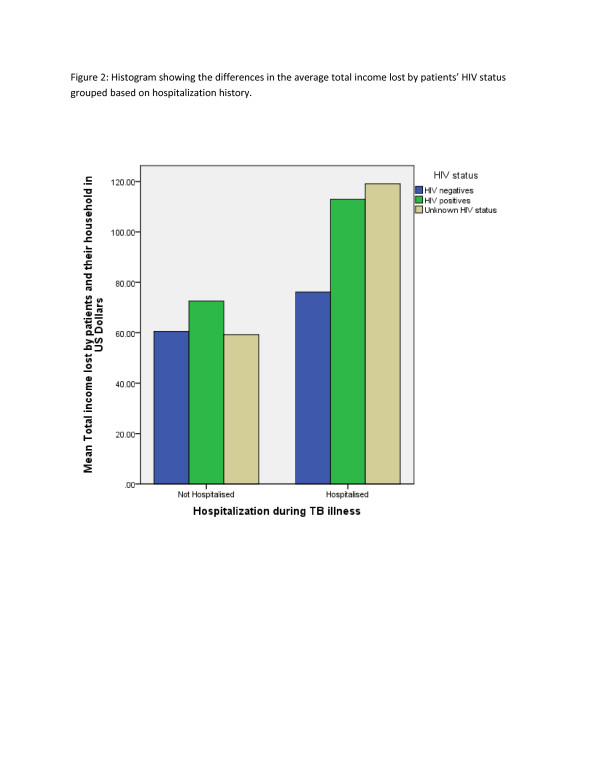
Histogram showing the differences in the average total income lost by patients’ HIV status grouped based on hospitalization history.

The income lost among the hospitalized group was estimated at $156/patient and about $114 in the non-hospitalized patients group (Table 2). The income lost varied by history of hospitalisation, gender and HIV status of the patients (Figures 1, 2).

**Table 2:** Income lost by hospitalization status

**Description**	**Hospitalized**	**Not hospitalized**
Average income lost by patient throughout the TB illness	$75.09	$69.62
Average income lost by other household members throughout the TB illness	$80.87	$43.89
**Total**	**$155.96**	**$113.51**

**Figure 3:**
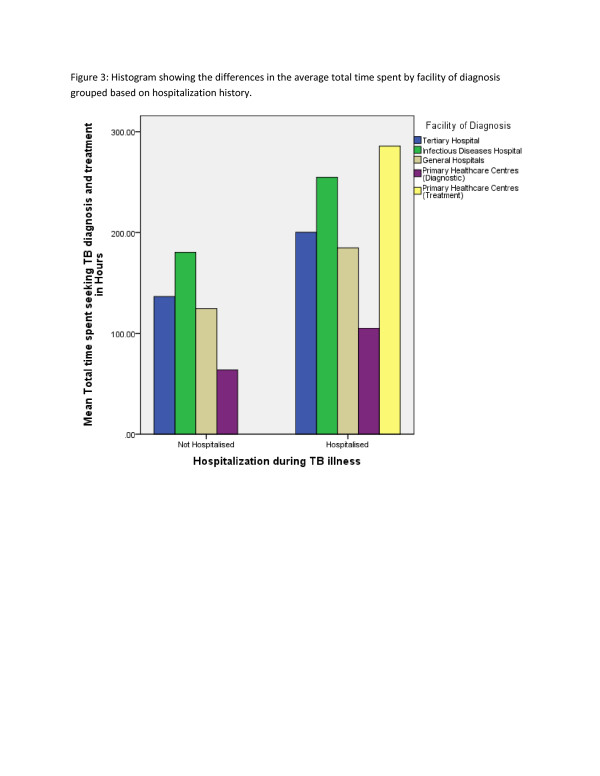
Histogram showing variations in the average total income lost and in the total time spent by patients’ occupation.

**Figure 4:**
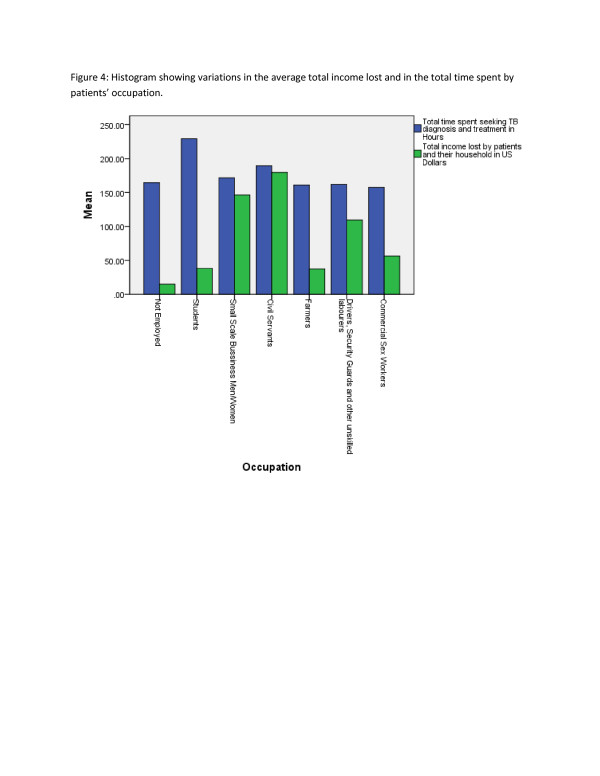
Histogram showing variations in the average total income lost and in the total time spent by patients’ educational attainment.

Univariate General Linear Model showed that age, gender, facility of diagnosis, level of education and occupation were statistically significant (p-values <0.05) predictors of the total (both patients and their households) income lost. However, AFB sputum-smear result and HIV status had no significant effects on the income lost (Table 3), (Figure3), (Figure4).

**Table 3:** Test of Between-Subject Effects (Univariate General Linear Model)

	**Total time spent by patients and households in Hours**			**Total Income lost by patients and households in US Dollars**		
	**df**	**F**	**p-value**	**df**	**F**	**p-value**
Age	36	1.268	0.158	36	1.673	0.015**
Gender	1	0.613	0.435	1	6.309	0.013**
Facility of Diagnosis	4	3.950	0.004**	4	2.873	0.024**
Sputum Smear test	1	1.687	0.195	1	2.793	0.096
Level of Education	5	0.510	0.769	5	4.459	<0.001**
HIV status	3	1.342	0.264	3	1.084	0.340
Occupation	6	0.681	0.665	6	6.268	<0.001**
History of Hospitalization	1	23.803	<0.001**	1	3.181	0.076

**Statistically significant effect.

### Time spent

Patients with history of hospitalisation during the TB illness spent an average time of 924.98 hours for seeking diagnosis and treatment whereas the non-hospitalised group spent an average of 141.29 hours. The estimated US dollar valued of these hours based on the US0.56/hour GNI based assumption was US517.98 and US$79.13 for hospitalised and non-hospitalised patient groups respectively (Table 4).

**Table 4:** Time spent in hours and value in US dollars by hospitalization status

**Description**	**Hospitalized**		**Not hospitalized**	
	**Av. Time spent**	**Value equivalent**	**Av. Time spent**	**Value equivalent**
Average time patients used for diagnosis and care throughout the TB illness	517.33hrs	$289.70	120.3hrs	$67.41
Average time spent by others on a TB patient throughout the TB illness	407.65hrs	$228.28	20.92hrs	$11.72
**TOTAL**	**924.98hrs**	**$517.98**	**141.29hrs**	**$79.13**

**Figure 5:**
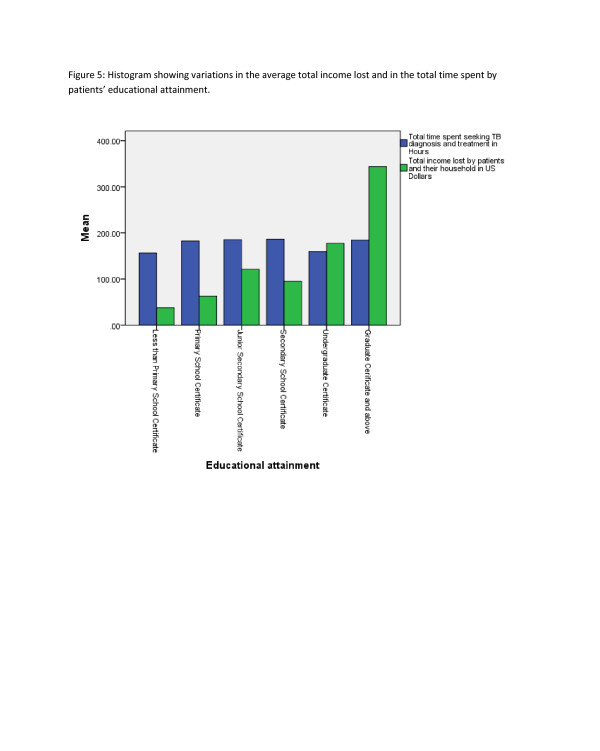
Histogram showing the differences in the average total time spent by facility of diagnosis grouped based on hospitalization history.

Hospitalisation and facility of diagnosis were statistically significant (p-value <0.05) associated with the total time (patients and household) spent on TB (Table 3) (Figure3), (Figure4), (Figure5).

## Discussion

The study estimated the average total income lost by TB patients and their household for the hospitalized and non-hospitalised patients groups at US156.96 and US$ 113.51 respectively. Lost in individual patient incomes did not varied much based on history of hospitalisation (US$75.09 vs. US$69.62 for the hospitalised and non-hospitalised patient groups respectively). However, average income lost to household members was observed to be much higher in the hospitalised patients group (US$80.87 vs. US$43.89 for the hospitalised and non-hospitalised patient groups respectively).

Age, gender, type of facility, level of education and occupation were found to be significant predictors of the total income lost (by patients and household) due to TB disease. AFB sputum-smear test result and hospitalisation were not significantly associated with the total income lost.

In this study, we also found that TB patients and their household spent an average of 924.98 hours in the hospitalised and 141.29 hours in the non-hospitalised patients groups seeking TB diagnosis and treatment. These times were valued at US$517.98 and US$79.13 for hospitalised and non-hospitalised patients respectively. Hospitalisation during the TB illness and the facility of diagnosis were found to be significant predictors of the total time spent. Age, gender, AFB sputum-smear results, level of education, HIV status and occupation were not significant predictors of the total time spent on TB illness.

Several studies have reported income lost due to Tuberculosis in some African countries. A study in Zambia reported an average of 48 days loss of income due to TB illness [8] and another study reported US$15.27 as the median total indirect cost of TB treatment in Zambia in 2006 [22]. Another study conducted in Dar es Salaam, Tanzania in 2002 reported a middle estimate of about US$431 as the household productivity lost due to Tuberculosis [16]. We found no study that reported the time spent by patients and their household members for seeking TB diagnosis and treatment services in any sub Saharan African country.

Considering the average annual income of TB patients in the study ($449.90 and $960.65 for female and male patients respectively) the income lost due to TB as found in this study could be described as catastrophic (more than 10% of the annual income [23]) to many patients and their households.

## Conclusion

Tuberculosis poses causes tremendous burden in terms of time and productivity lost to both patients and their households which could be catastrophic to many patients and their families whom are mostly impoverished and economically very vulnerable.

## Competing interests

The authors declare that they have no competing interests.

## Authors’ contributions

All authors were involved in the conceptualization of the study. NU participated in the data collection, data entry and did the data analysis. NU drafted the manuscript while RF, IA, MB reviewed and subsequently improved the manuscript. All authors read and approved the final manuscript.
